# Recognition of *Orobanche cumana* Below-Ground Parasitism Through Physiological and Hyper Spectral Measurements in Sunflower (*Helianthus annuus* L.)

**DOI:** 10.3389/fpls.2017.00909

**Published:** 2017-06-07

**Authors:** Amnon Cochavi, Tal Rapaport, Tania Gendler, Arnon Karnieli, Hanan Eizenberg, Shimon Rachmilevitch, Jhonathan E. Ephrath

**Affiliations:** ^1^The French Associates Institute for Agriculture and Biotechnology of Drylands, The Jacob Blaustein Institutes for Desert Research, Ben-Gurion University of the NegevBeer-Sheva, Israel; ^2^The Remote Sensing Laboratory, The Swiss Institute for Dryland Environmental & Energy Research, The Jacob Blaustein Institutes for Desert Research, Ben Gurion University of the NegevBeer-Sheva, Israel; ^3^Department of Plant Pathology and Weed Research, Newe Ya’ar Research Center, Agricultural Research Organization, Volcani CenterRamat Yishay, Israel

**Keywords:** broomrape, early attachment, mesophyll, PLS-R, minerals content

## Abstract

Broomrape (*Orobanche* and *Phelipanche* spp.) parasitism is a severe problem in many crops worldwide, including in the Mediterranean basin. Most of the damage occurs during the sub-soil developmental stage of the parasite, by the time the parasite emerges from the ground, damage to the crop has already been done. One feasible method for sensing early, below-ground parasitism is through physiological measurements, which provide preliminary indications of slight changes in plant vitality and productivity. However, a complete physiological field survey is slow, costly and requires skilled manpower. In recent decades, visible to-shortwave infrared (VIS-SWIR) hyperspectral tools have exhibited great potential for faster, cheaper, simpler and non-destructive tracking of physiological changes. The advantage of VIS-SWIR is even greater when narrow-band signatures are analyzed with an advanced statistical technique, like a partial least squares regression (PLS-R). The technique can pinpoint the most physiologically sensitive wavebands across an entire spectrum, even in the presence of high levels of noise and collinearity. The current study evaluated a method for early detection of *Orobanche cumana* parasitism in sunflower that combines plant physiology, hyperspectral readings and PLS-R. Seeds of susceptible and resistant *O. cumana* sunflower varieties were planted in infested (15 mg kg^-1^ seeds) and non-infested soil. The plants were examined weekly to detect any physiological or structural changes; the examinations were accompanied by hyperspectral readings. During the early stage of the parasitism, significant differences between infected and non-infected sunflower plants were found in the reflectance of near and shortwave infrared areas. Physiological measurements revealed no differences between treatments until *O. cumana* inflorescences emerged. However, levels of several macro- and microelements tended to decrease during the early stage of *O. cumana* parasitism. Analysis of leaf cross-sections revealed differences in range and in mesophyll structure as a result of different levels of nutrients in sunflower plants, manifesting the presence of *O. cumana* infections. The findings of an advanced PLS-R analysis emphasized the correlation between specific reflectance changes in the SWIR range and levels of various nutrients in sunflower plants. This work demonstrates potential for the early detection of *O. cumana* parasitism on sunflower roots using hyperspectral tools.

## Introduction

For many years, the underground development of root parasitic plants has been a mystery for both researchers and farmers. Broomrape parasitism of host crop roots is generally recognized only when parasite inflorescences break through the soil surface, at which point irreversible damage has already been done to the crop (Mauromicale et al., 2008; Longo et al., 2010). Over time, we have learned more about the below-ground developmental stages of parasitic plants (Heide-Jørgensen, 2013) and that the germination of parasitic seeds is triggered by specific hormonal signals in the root exudates of host plants (Yoneyama et al., 2010). After recognition, the parasite seed germinates and the rootlet grows toward the host’s roots. Finally, the parasite penetrates the host’s roots and forms a vascular connection with the host (Yoneyama et al., 2013). The parasite then starts to exploit the host’s water, mineral and energy resources, slowly accumulating biomass in preparation for its final stage of above-ground inflorescence (Westwood, 2013). In general, more severe infestations will lead to greater crop damage (Grenz et al., 2008).

Several methods have been developed over the years to reduce damage to sunflower (*Helianthus annuus* L.) caused by *Orobanche cumana* Waller, Sunflower Broomrape (Orobanchaceae family). Castejon-Munoz et al. (1993) showed that earlier sowing dates reduces parasite-induced damage to sunflowers. However, the utility of that method is limited, due to yield reductions caused by shifting sowing dates earlier into a period of cooler weather. Another approach for managing *O. cumana* parasitism is the development of resistant cultivars. However, such resistance is temporary and, after several growth cycles, a new strain of broomrape is usually able to parasitize the crop (Rubiales et al., 2009). A different approach to parasite management is the application of low rates of herbicides, which can reduce parasite damage in many crops (Eizenberg et al., 2012a; Cochavi et al., 2015). In practice, as the herbicide is transported from the foliage through the host vessels down to the parasite’s tubercle, the parasite is injured (Eizenberg et al., 2012a). In this context, the rate and timing of herbicide applications to host foliage are crucial for optimal parasite management (Eizenberg et al., 2006); inaccurate herbicide application can lead to crop damage caused by the untreated parasite or too high doses of herbicide.

To predict the timing of the underground development of *O. cumana*, a thermal model was constructed (Eizenberg et al., 2012b). Using this model, a low rate of imazapic (an acetolactate synthase inhibitor) applied at the tubercle stage of parasite development provides highly effective control. Despite the efficacy of this herbicide, the thermal model predicts parasite development based on uniform spatial distribution of the parasite in the field, i.e., that essentially all host plants in the field are infected. Therefore, even non-parasitized plants are sprayed with herbicide, which can lead to unnecessary herbicide use and crop injury.

The use of physiological measurements can provide early evidence of parasite attachment to host roots. The advantage of physiological measurements lies in their ability to highlight changes in plant status before those changes become visible (Levitt, 1980). Nevertheless, to date, substantial broomrape-related physiological changes have been reported only after the parasite’s inflorescence has already appeared above ground level (Barker et al., 1996; Alcántara et al., 2006), at which point herbicide applications are virtually ineffective in improving crop yield. Moreover, physiological measurements often require proficient manpower and intrusive instruments and may prove to be time- and cost-ineffective, due to biotic and abiotic variability in the field. A plausible solution to this latter issue is the use of hyperspectral remote-sensing instruments, which offer a faster, cheaper and non-destructive way to assess crop physiology (Mulla, 2013). Specifically, narrow-band visible (VIS, 350–700 nm) to shortwave infrared (SWIR, 1400–1700 nm) sensors are suitable for this purpose; they are sensitive enough to monitor slight changes in various biochemical properties (Blackburn, 2007).

Over the years, variations in numerous physiological properties have been spectrally monitored with such equipment, including changes in leaf water balance (Dzikiti et al., 2010), nutrient status (Menesatti et al., 2010), pigment content (Garrity et al., 2011), xanthophyll cycle activity and fluorescence (Rapaport et al., 2015) and more (Mulla, 2013). In the context of parasites, a previous study found changes in the reflectance of *O. cumana*-infected sunflower leaves illuminated by UV light (Ortiz-Bustos et al., 2016). Researchers found differences in the reflectance of wavelengths of 680 and 740 nm, which were related to changes in chlorophyll content and functioning.

Despite the potential advantages of hyperspectral signatures, narrow-band datasets tend to be highly noisy and therefore require the use of dimensionality-reduction techniques (Mariotto et al., 2013). Specifically, since only a few principal wavelengths of the entire VIS-SWIR spectrum are strongly correlated with physiological variables, whilst the rest are actually redundant – negatively affecting the derivation of indices – the use of optimal wavelength-selection processes is essential (Wold et al., 2001). This issue can be addressed by employing advanced statistical techniques, such as the partial least squares regression (PLS-R) method (Wold et al., 2001). That method was previously shown to be effective for highlighting the most physiologically sensitive wavebands and producing reliable spectral models (e.g., Rapaport et al., 2015). In practice, the PLS-R technique can be used to correlate collinear, noisy and distribution-free datasets, even when the number of predictors greatly exceeds the number of predictions – making it very suitable for dealing with hyperspectral signatures. Geladi and Kowalski (1986) demonstrated the superiority of targeted indices over traditional and broad-band spectral models.

This study focuses on the relationship between *O. cumana* parasitism and the reflectance of the host sunflower leaves. During the study, susceptible and resistant plants were examined, in order to evaluate the effect of *O. cumana* parasitism on different varieties of sunflowers. Physiological, anatomical, and chemical measurements of plant foliage were simultaneously taken. Statistical analysis, using commercially available software, was then applied to the measurements, to determine whether correlations exist between specific wavelengths and physiological, structural, and chemical changes in the leaf.

## Materials and Methods

### Plant Material and Growth Conditions

Sunflower seeds (cv. D. Y. 3, which is *O. cumana*-susceptible, and cv. Emek 3, which is *O. cumana*-resistant; Sha’ar Ha’amakim Seeds, Sha’ar Ha’amakim, Israel) were sown. The seeds were planted in 4-L pots filled with infested or non-infested soil (Newe Ya’ar soil, Chromic Haploxererts, fine clayey, montmorillonitic, thermic, 55% clay, 25% silt and 20% sand, 2% organic matter and pH 7.2). Parasitic *O. cumana* seeds were brought from the Newe Ya’ar Research Center’s collection. These seeds were collected in 2010 from Havat Gadash, Hahula Valley, Israel. The seeds were passed through a 300-mesh sieve and stored in the dark at 4°C. A germination test was performed under standard conditions at 25°C, with germination stimulated by the synthetic stimulant GR-24 (Yoneyama et al., 2013), which is commonly used for broomrape germination tests. The GR-24 was applied at a rate of 10 mg kg^-1^ after 12 days of pre-conditioning, resulting in a germination rate of 84%. Parasite seeds were mixed into the soil using a 50 L cement mixer, creating a concentration of 15 mg kg^-1^. Over the course of the experiment, soil temperature was monitored using temperature data-loggers (UA-001-08 data logger, Onset, Co., Bourne, MA, United States) buried at a depth of 10 cm. The parasite’s below-ground attachment was estimated using the thermal model developed by Eizenberg et al. (2012b). Over the course of the experiment, parasite development was also monitored visually by selecting pots, gently removing soil from the sunflower plants, washing their roots and examining any parasite development, as described by Eizenberg et al. (2006). Measurements were conducted as follows: the first measurements after two true leaves developed [23 days after planting (DAP)]; the second measurements after *O. cumana* tubercles were established on the host roots (31 DAP); the third measurement after 63% of *O. cumana* tubercles were established on the host roots (36 DAP); and the last measurement after *O. cumana* inflorescences emerged above the ground level (51 DAP). Pots were opened in order to validate *O. cumana* development according to the model. Each treatment contained 23 pots, with eight used for each of the measurements.

Plants were grown in a semi-controlled greenhouse (Midreshet Ben-Gurion, Ramat Hanegev, Israel) in which temperatures ranged between 20 and 30°C, with a radiation level of 800 μmol photons m^-2^ s^-1^ at midday. Plants were irrigated using 2 l h^-1^ drippers, for field capacity plus 10% in order to remove salts from the upper soil level. Run off water was monitored in order to evaluate the water required by the plants in the different treatments. Fertilizer (27:10:17 N-P-K) was applied through the drip-irrigation system at a rate of 1 g fertilizer/500 L water. Fertilizer application began 14 DAP, after the sunflower plants had begun to develop true leaves.

### Spectral Measurements

Adaxial hyperspectral radiance was measured with a Pro-FR Field Spectrometer (Analytical Spectral Devices, Inc., Boulder, CO, United States) coupled to an 1800-12S Integrating Sphere (Li-Cor, Inc., Lincoln, NE, United States). The signatures were collected within the 350–2500 nm spectrum at a resolution of 1 nm and were normalized into reflectance units by frequently taking supplementary readings of BaSO_4_ tablets. Measurements were taken from the youngest fully matured leaf, three measurements from each leaf and total of eight replicates, at midday. During the same period, physiological measurements were taken under clear, cloudless skies.

### Physiological Measurements

Physiological measurements were taken starting after the formation of two true leaves (23 DAP); each of the measurements included eight replicates. All measurements were taken from the youngest fully matured leaf. Photosynthesis and transpiration rate, stomatal conductance, and vapor pressure deficit (VPD) were measured with a portable infra-red gas analyzer (IRGA) system (Li-6400, Li-Cor, Lincoln, NB, United States). Non-photochemical quenching (NPQ) was calculated based on the maximal fluorescence of the youngest fully matured leaf in the light (F_m_′) and dark-adapted (F_m_, 30 min adaptation time) as shown in Eq. (1):

(1) $$\frac{{\text{F}}_{\text{m}}{\text{-F}}_{\text{m}}{}_{\text{'}}}{{\text{F}}_{\text{m}}{}_{\text{'}}}$$

(Bilger and Bjorkman, 1990), using a Mini-PAM I (Walz, Effeltrich, Germany) photosynthesis yield analyzer. Relative water content was measured according to Smart and Bingham (1974); for each measurement, the youngest fully matured leaves were taken from four plants from each treatment. Chlorophyll content was measured as described by Bruinsma (1963): chlorophyll was extracted from leaf disks by soaking the disks overnight in dimethyl sulfoxide (DMSO), followed by spectrophotometer absorption measurements at wavelengths of 665 and 649 for chlorophyll *a* and *b* content evaluations, respectively.

### Determination of Leaf Mineral Content

After physiological measurements were taken, the youngest fully matured leaf samples were taken for mineral analysis and oven-dried for 48 h in 65°C. Carbon, nitrogen, and hydrogen levels were measured using a FLASH 2000 CHNS/O Analyzer (Thermo Fisher Scientific, Waltham, MA, United States). Additional mineral measurements were taken using Inductively Coupled Plasma Optical Emission Spectroscopy (ICP-OES, Varian 720 ES, Agilent Technologies, Santa Clara, CA, United States). Dry plant material was extracted by overnight ash in a 470°C oven, followed by an extraction using nitric acid. Samples were diluted with double-distilled water before measurements were taken. At each time point, mineral measurements involving five replications per treatment were taken.

### Anatomic Analysis

Leaf tissue sections were taken from all treatments (susceptible and resistance cultivar, infected and non-infected plants) from the youngest fully matured leaf. Samples were taken from the widest part of the leaf without the central vein. Cross-sections were prepared as described by Rewald et al. (2012) and Rapaport et al. (2014). Digital images of the cross-sections were then taken with an Axio Imager A1 Light Microscope (Carl Zeiss Microscopy, Inc., TH, Germany) and 6 × 100 magnification images were produced using its AxioVision 4.6.3 software. Cross-sections were analyzed using Digimizer software (version 4.5.2, MedCalc Software, Ostend, Belgium). Measured parameters included tissue width and air space capacity. Air-space volume was defined as the proportion of the air spaces of the total leaf area. To evaluate air-space capacity in a uniform manner, analyzed leaf sections were chosen specifically for minimal presence of leaf vessels within a fixed area (300 μm × 300 μm square).

### Statistical Analysis

The experiment was utilized a random design with eight replications and analyzed using one-way ANOVA. Means were compared with Tukey’s HSD test, using JMP software (vers. 7, SAS, Inc., Cary, NC, United States). Correlations between physiological measurements and specific wavelength changes were calculated using PLS-R (PLS_Toolbox 7.03 software, Eigenvector Research, Inc., Manson, WA, United States) run in a MATLAB 7.12 environment (Mathworks, Inc., Natick, MA, United States), as explained in Rapaport et al. (2015). In short, (1) the independent (wavelengths) and dependent (physiology) variables were mean-centered during the pre-processing stage; (2) a PLS-R model was then built, and the contribution of all wavelengths was assessed using the variable importance in projection (VIP) statistic; (3) uncorrelated wavebands were then iteratively removed, to improve the model’s accuracy; and (4) once no more areas for improvement were found, the robustness of the best model and of the most physiologically sensitive were assessed by considering the latent variable (LV) amount and the ability to explain the variation in the datasets. All the parameters were tested twice, once separately (physiological separately, minerals content, and anatomic cross-section separately) and after that in one experiment. Therefore only the second experiment is presented.

## Results

### Spectral Measurements

**FIGURE 1:**
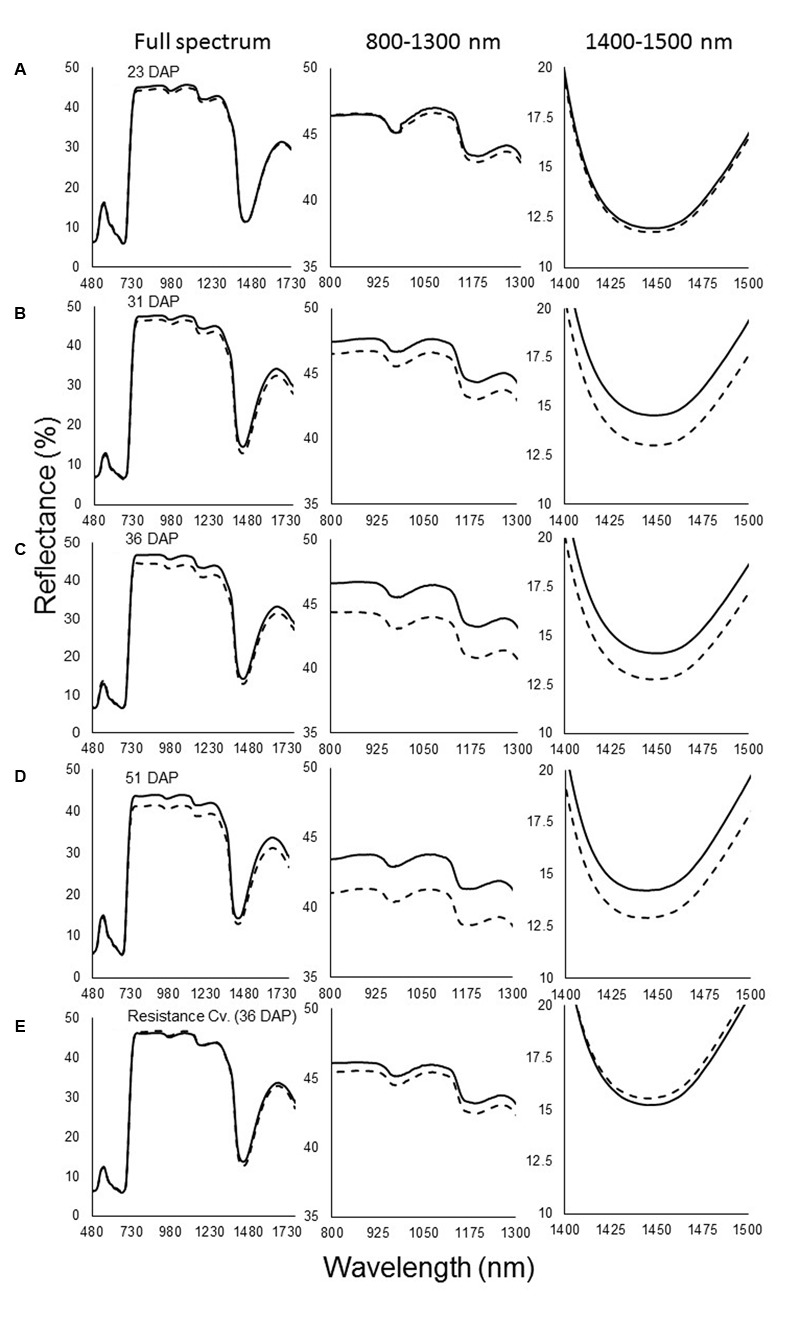
Spectral signature of reflectance of non-infested (—) and infested (—) susceptible sunflower leaves. **(A)** Measurements taken at 23 days after planting (DAP), before *Orobanche cumana* tubercles became established; **(B)** 31 DAP, first attachment of *O. cumana*; **(C)** 36 DAP, 63% of all *O. cumana* attachments were established on the sunflower roots; **(D)** 51 DAP, *O. cumana* inflorescences emerged. **(E)** Resistance cultivar 36 days after planting. Lines represent means of eight plants; three measurements were made on each plant.

The spectral signatures of the susceptible sunflower leaves indicated that *O. cumana* parasitism mainly affects reflectance in the far-NIR and SWIR wavebands. The first measurement was taken at 23 DAP and, at that time, no differences were seen between infested and non-infested pots (**Figure 1A**). At the next measurement (31 DAP), after *O. cumana* attachment appear on the host roots, differences in the reflectance between infected and uninfected susceptible plants were noted. These differences were pronounced in the far-NIR waveband, between 800 and 1300 nm. The reflectance level of the leaves of sunflower plants infected with *O. cumana* was found to be 1% lower in that range. Additional differences were found in the 1400–1500 nm range, wherein the reflectance of plants infected with *O. cumana* decreased by 1.5% (**Figure 1B**). The next measurement (36 DAP) conducted after 63% of the tubercle appear on the host roots according to the model, the reflectance differences between treatments were greater in the far-NIR range, but not in the SWIR range (∼2% between treatments; **Figure 1C**). Furthermore, after the emergence of *O. cumana* inflorescences, the gap between treatments in the NIR area remained the same as they had been during the earlier measurements (**Figure 1D**).

**FIGURE 2:**
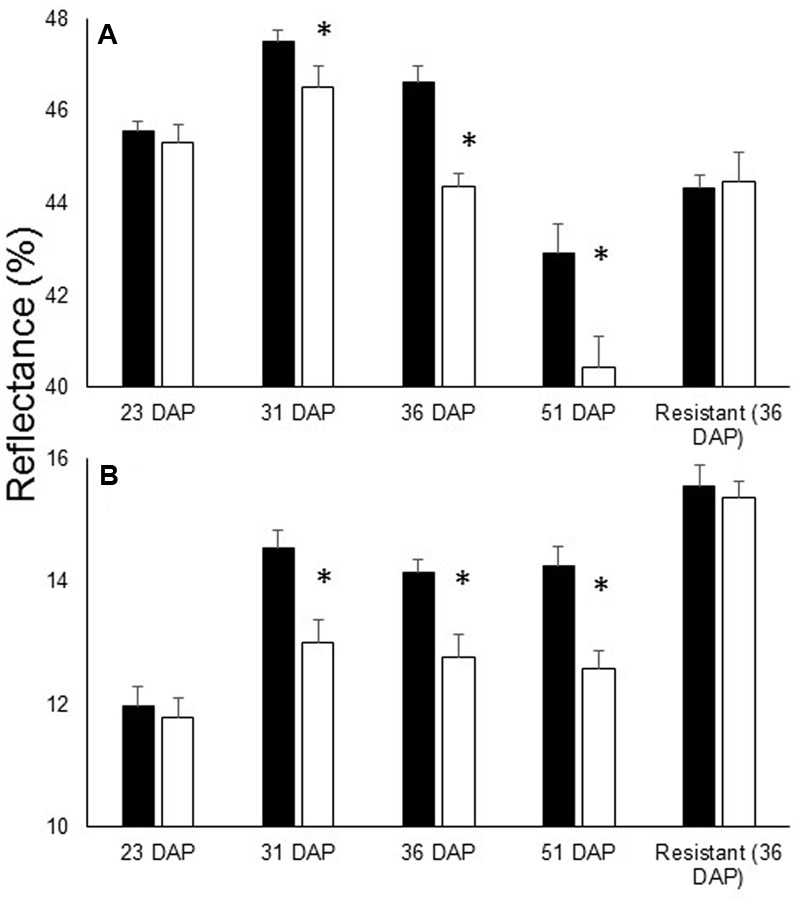
Leaf reflectance of specific wavelengths in susceptible cultivar 23, 31, 36, and 51 DAP in addition to the reflectance of the resistant cultivar 36 DAP. **(A)** Average reflectance in the far-NIR (800–1300 nm) and **(B)** SWIR (1450 nm range). *O. cumana* attachments first observed at 31 DAP. There were eight replicates for each treatment. Bars refer to SE of the mean and asterisks indicate significant differences between treatments (0.05 ≥ α).

During the experiment, no *O. cumana* development on the resistant cultivar roots was observed. Examination of the reflectance of the leaves of plants of the resistant cultivar revealed no differences between treatments throughout the experiment (i.e., whether in infested and non-infested soil) (**Figure 1E**). Statistical analysis indicated that the differences in the far-NIR and SWIR areas after *O. cumana* attachment are significant (**Figure 2**).

### Physiological Measurements

Physiological measurements taken over the course of the experiment indicated no significant differences at the early attachment stage. No statistically significant differences were observed in the carbon assimilation, stomatal conductance, and transpiration rate of the parasitized plants compared with those of the control plants at any of the stages of parasite development (23–51 DAP). NPQ measurements also revealed no differences over the course of the experiment. Relative water content was similar in the infected and non-infected plants. Chlorophyll content did not show any significant differences until the *O*. *cumana* inflorescences emerged (**Table 1**). During the experiment, no physiological differences were found in the resistant cultivar between infested and non-infested pots.

**Table 1:** Physiological measurements of infected and non-infected susceptible sunflower plants.

Treatment/DAP		23	31	36	51
Carbon assimilation (μmol m^-2^ s^-1^)	Control	11.29 ± 0.66l	12.20 ± 0.74	14.53 ± 0.80	11.74 ± 1.94
	*O. cumana* infected	13.22 ± 0.84l	11.27 ± 1.05	10.86 ± 1.48	9.76 ± 1.30
Stomatal conductance (mol m^-2^ s^-1^)	Control	0.40 ± 0.02 l	0.30 ± 0.02	0.41 ± 0.04	0.24 ± 0.06
	*O. cumana* infected	0.37 ± 0.04 l	0.23 ± 0.02	0.29 ± 0.06	0.19 ± 0.03
Transpiration (mmol m^-2^ s^-1^)	Control	6.74 ± 0.21 l	4.79 ± 0.34	6.56 ± 0.46	4.12 ± 1.00
	*O. cumana* infected	6.28 ± 0.40 l	3.8 ± 0.30	4.78 ± 0.76	3.38 ± 0.46
Non-photochemical quenching	Control	2.46 ± 0.22 l	2.32 ± 0.17	2.72 ± 0.26	2.16 ± 0.15
	*O. cumana* infected	2.12 ± 0.16 l	2.52 ± 0.86	2.63 ± 0.39	2.32 ± 0.20
Relative water content (%)	Control	75.61 ± 2.42l	79.35 ± 5.01	86.84 ± 7.50	92.67 ± 3.83
	*O. cumana* infected	72.79 ± 1.41l	79.31 ± 1.07	85.44 ± 4.30	91.33 ± 2.12
Chlorophyll content (mg l^-1^)	Control	0.03 ± 0.002l	0.04 ± 0.004	0.04 ± 0.003	0.04 ± 0.002
	*O. cumana* infected	0.03 ± 0.001l	0.04 ± 0.002	0.04 ± 0.001	0.03 ± 0.001

*Orobanche cumana tubercles appear first in measurements done 31 DAP. Carbon assimilation, stomatal conductance, transpiration, and non-photochemical quenching include eight replications; relative water content, and chlorophyll content include four replications. Means comparison was done using one -way analysis of variance with Student’s *t*-test (α < 0.05). The result represents the mean ± SE.*

### Mineral Evaluations

**FIGURE 3:**
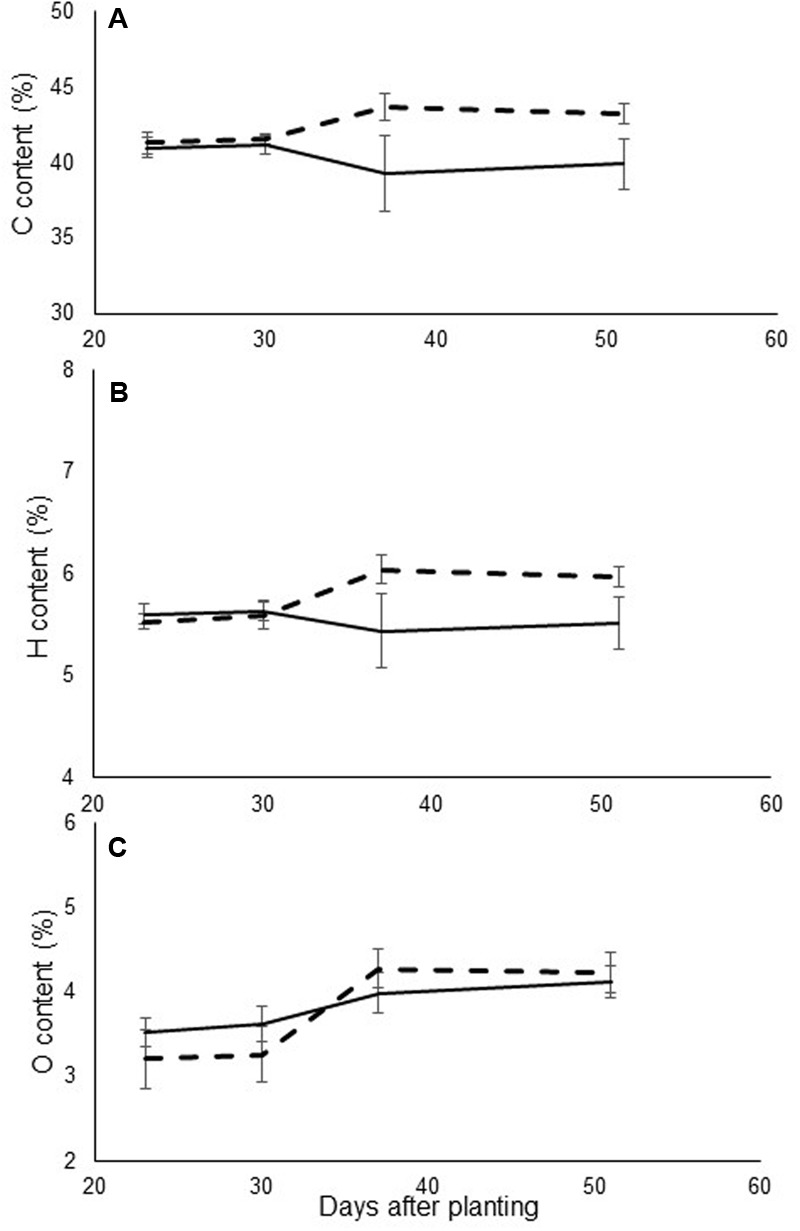
**(A)** Carbon, **(B)** hydrogen, and **(C)** nitrogen content (% of total dry weight) of leaves in control (continuous line) and in *O. cumana*-infected (dashed line) susceptible sunflower plants. *O. cumana* infections appeared on plant roots 31 DAP. There were five replicates of each measurement. Bars represent standard errors of mean.

Evaluations of leaf mineral content revealed differences in carbon and hydrogen values. Mineral contents were higher in the infected plants, as compared to the non-infected ones (**Figure 3**). However, no consistent pattern of differences in nitrogen content was observed among the different treatments.

**FIGURE 4:**
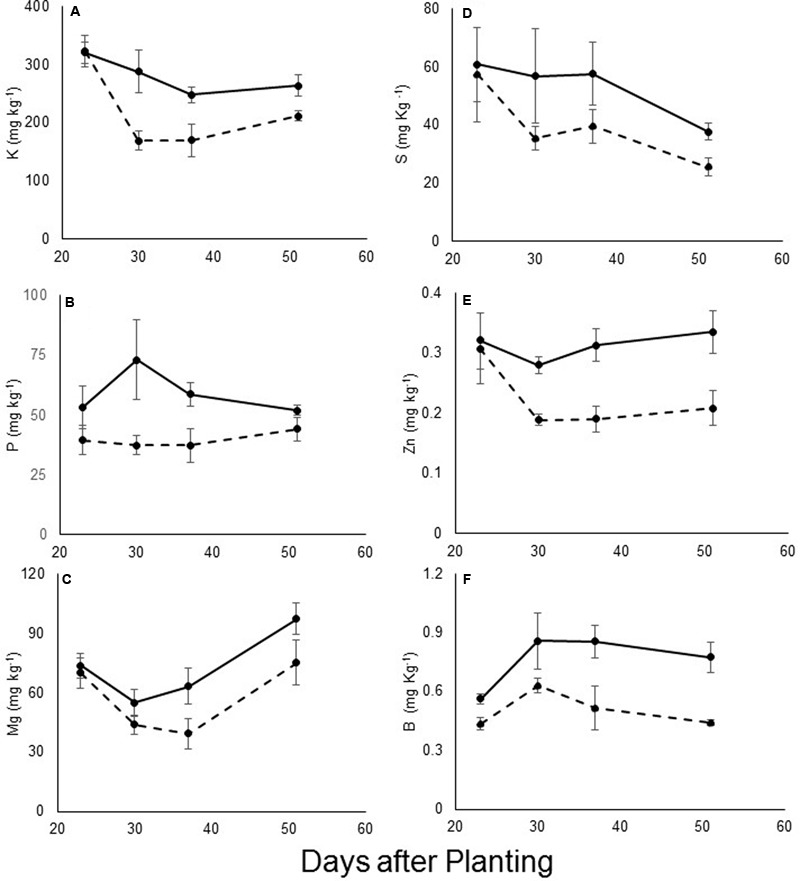
**(A)** Phosphorus, **(B)** potassium, **(C)** magnesium, **(D)** sulfur, **(E)** zinc, and **(F)** boron content (mg kg^-1^) of leaves of control (—) and *O. cumana*-infected (—) susceptible sunflower plants. *O. cumana* infections were first observed on plant roots 31 DAP. There were five replicates of each measurement. Bars represent standard errors of mean.

The levels of other minerals were measured using ICP technology. Significant differences were found in the levels of the macro-elements potassium, phosphorus, magnesium, and sulfur between the infected and non-infected plants during the early stage of parasite development (**Figures 4A–D**). By contrast, the calcium content did not vary between the treatments (data not shown). Differences were noted in the levels of the microelements zinc and boron (**Figures 4E,F**, respectively). For all of these elements, the differences were more pronounced at the early attachment stage (starting at 31 DAP). The levels of other elements such as chlorine, sodium, iron and manganese remained similar between treatments, and changed only after the emergence of the parasite (data not shown). No mineral content differences were found in the resistant cultivar between infested and non-infested pots at any time during the experiment.

### Anatomical Analysis of Leaf Cross-Sections

**FIGURE 5:**
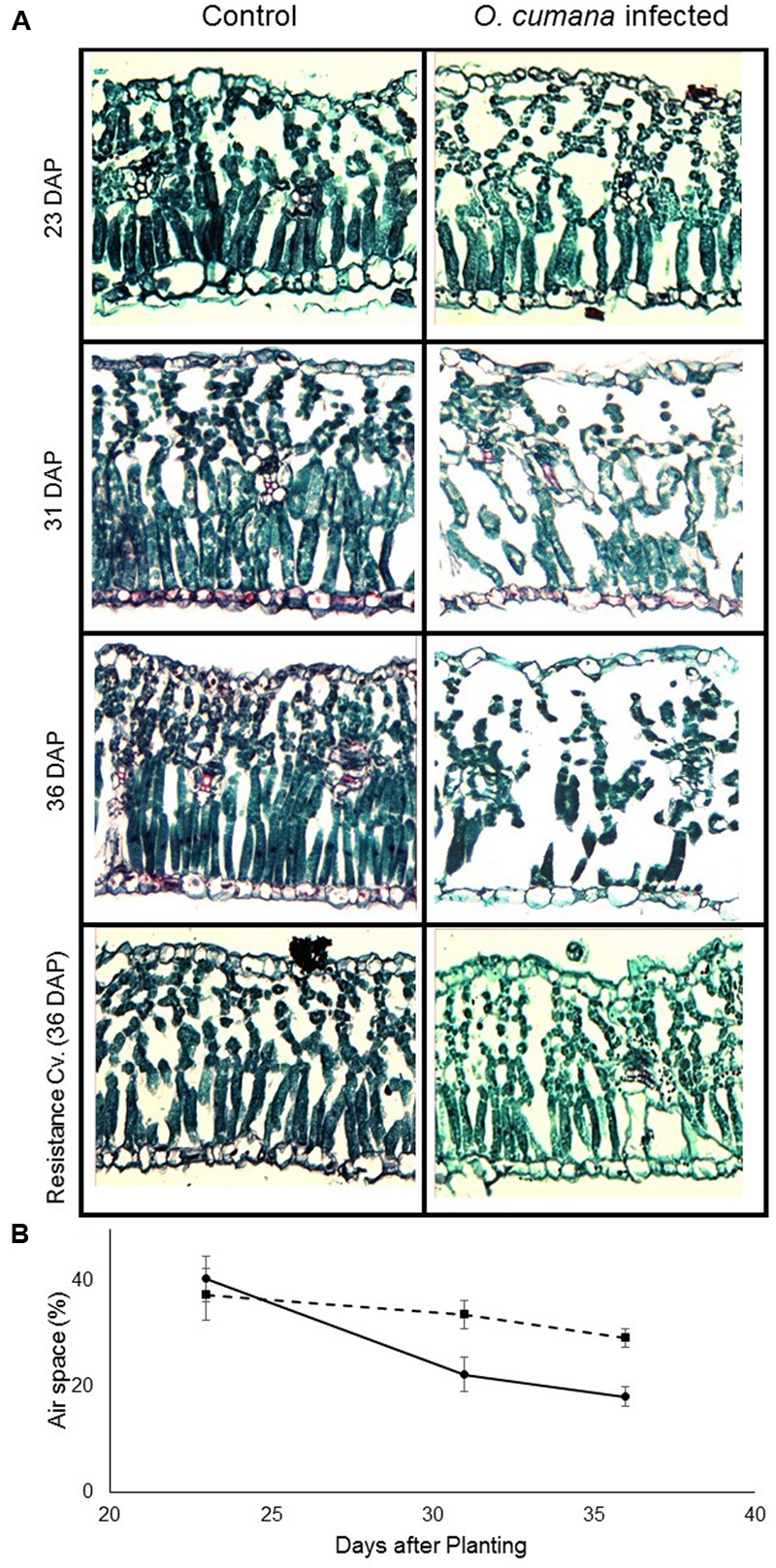
**(A)** Sections of the youngest fully mature susceptible (23, 31, and 36 DAP) and resistant (36 DAP) sunflower leaves of control (left) and *O. cumana-*infested (right) sunflower plants. *O. cumana* tubercles were found starting from 31 DAP; no *O. cumana* attachment were found on the resistance cultivar roots. **(B)** Air spaces in non-infected (—) and *O. cumana-*infected (—) sunflower leaves. The relative amount of air space was calculated as the proportion of the total section area taken up by air space. There were four replicates for each treatment. Bars indicate standard errors of mean.

On the first sampling date, no differences were found between the leaves of the control sunflower plants and those infected with *O. cumana*. In both treatments, relative air-space capacity, in both the spongy and the palisade mesophyll, was about 40%. However, a short time after the first *O. cumana* attachments appeared, differences were noted between treatments (**Figure 5**). The relative air-space capacity in the mesophyll of non-infected plants was about 20% on the following two sampling dates. By contrast, leaf cross-sections of *O. cumana-*infected plants exhibited air space capacity of about 30% (**Figure 5B**). Among the leaves of the resistant cultivar, no differences between infected and non-infected plants were observed over the course of the experiment, therefore data not shown.

### PLS-R Analysis

**FIGURE 6:**
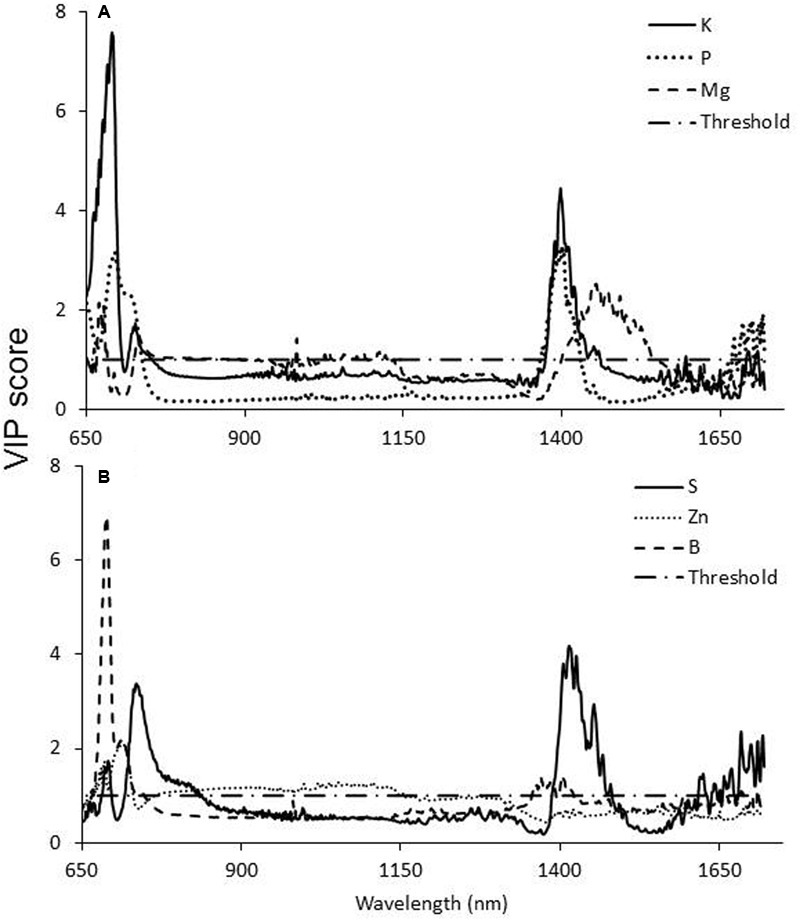
Variable importance in projection (VIP) scores of potassium phosphorus and magnesium **(A)** and sulfur, zinc, and boron **(B)** content in susceptible sunflower leaves according to the PLS-R analysis. Values higher than the threshold (>1) indicate a significant contribution of the specific wavelength to the overall variance.

Statistical analysis revealed a strong correlation between the levels of different nutrients in the leaves and the reflectance of specific wavelengths (**Figure 6** and **Table 2**). VIP statistics revealed the shared importance of the wavelengths in both the VIS (primarily around 675 nm) and SWIR (mainly around 1400–1500 nm) wavebands. Specifically, element-correlated wavelengths were found at 685 nm for potassium; 700 and 1400 nm for phosphorus; 690, 1000, and 1450 nm for magnesium (**Figure 6A**); 670 and 1450 nm for sulfur; and 690 and 1400 nm for boron. However, zinc-correlated wavelength VIP scores were lower (**Figure 6B**).

**Table 2:** Evaluation of different elements partial least squares regression (PLS-R) models.

Element	Explained variance (%)	*R*^2^	RMSEC	Q_3_-Q_1_	RPIQ
K	75.18	0.74	54.41	329.10	6.05
P	38.68	0.36	16.61	112.09	6.75
Mg	79.44	0.73	13.85	98.43	7.11
Zn	72.86	0.69	0.07	0.38	5.28
S	63.97	0.52	17.55	103.63	5.90
B	41.9	0.42	0.17	1.03	6.15

*Each model was built from 40 observations (five replicates from two treatments over four time points), and include six latent variables. Q_3_-Q_1_, maximal element range between samples; RPIQ, (Q_3_-Q_1_)/RMSEC. Mineral contents are in mg Kg^-1^.*

**FIGURE 7:**
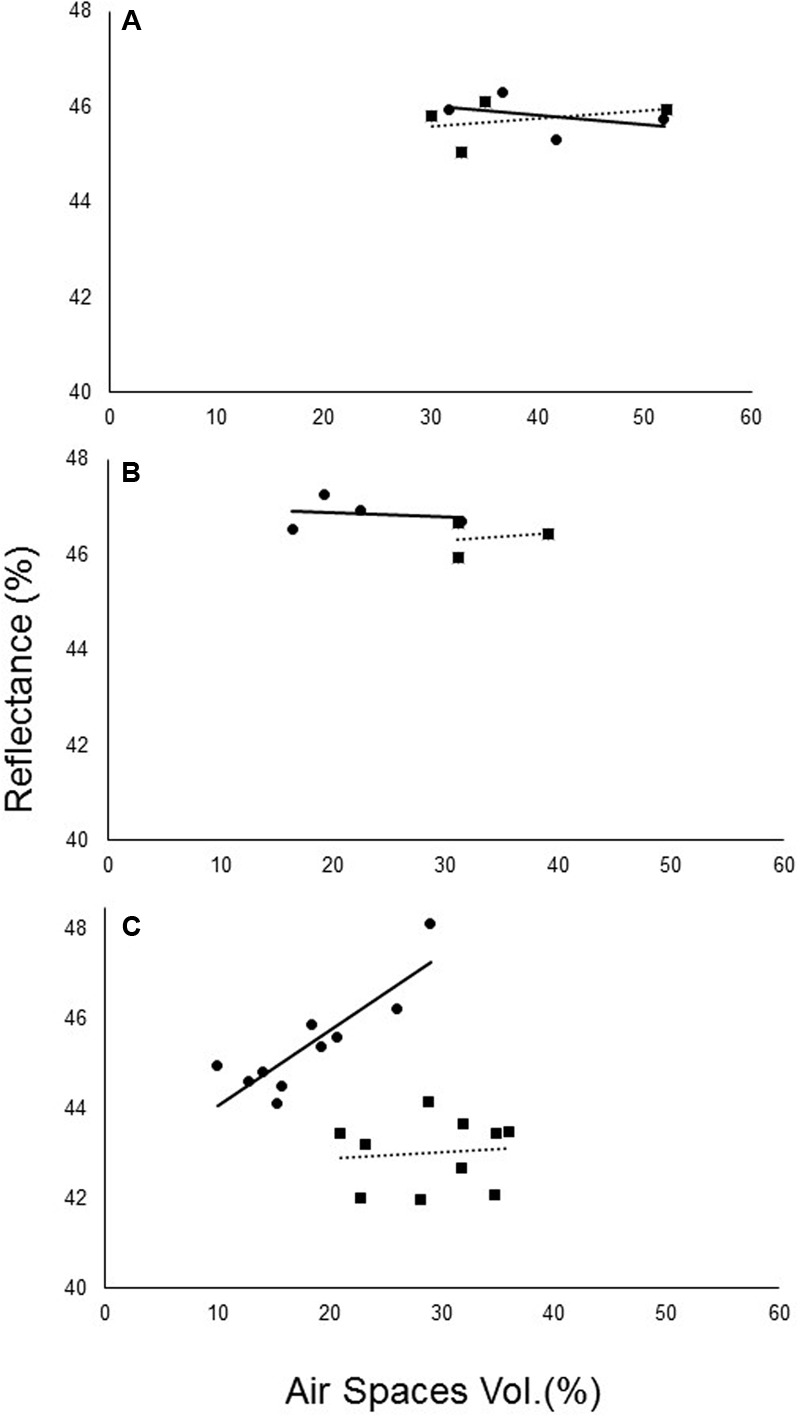
Correlation between non-infected (—) and *O. cumana-*infected (—) air spaces in sunflower leaves and the average spectral reflectance at 800–1300 nm. **(A)** Before *O. cumana* attachment (23 DAP); **(B)** when *O. cumana* attachment was first observed (31 DAP); **(C)** 36 DAP in susceptible sunflower leaves.

Reflectance at the chosen wavelengths and the relative size of air spaces in the leaf cross-sections were highly correlated. Before the parasite attached, no significant differences in relative air-space capacity were noted between the treatments (**Figure 7A**). However, after the attachment of *O. cumana* to the sunflower roots, significant differences in air-space capacity were noted (**Figures 7B,C**).

## Discussion

Over the years, many options have been suggested for the management of root parasites. The timing for applying herbicides is crucial; they must be applied when the parasite is in its early developmental stage underground. However, the spatial distribution of parasites in the field are not considered when applying the herbicides. Hence, unnecessary herbicide applications are common. In this study, a non-destructive method detecting early parasitism was developed.

Hyperspectral measurements of leaves of infected and non-infected susceptible sunflower plants revealed that the first parasite attachments causes changes in the VIS-SWIR reflectance signature of sunflower leaves. Unlike other stress responses, which have been found to induce changes in the VIS range (350–700 nm; Rapaport et al., 2015), no changes in this range were noted in this study. Nonetheless, changes were detected in the NIR and SWIR ranges. Thus, the difference in the reflectance of the different treatments was greater on the following measurement time-points. These results are somewhat similar to those of Ortiz-Bustos et al. (2016), who demonstrated that differences in the leaf fluorescence of *O. cumana*-infected vs. non-infected sunflower plants are pronounced at 680 and 740 nm after UV light induction. By using UV-induced multicolor fluorescence imaging, the researcher found differences in the wavebands fluorescence. In this experiment, we measured the reflectance of a broad spectrum of wavelengths and noted differences at additional wavelengths in the non-visible light spectrum. The reflectance difference between treatments are thought to be related primarily to tissue structure and water absorption in the leaf (Carter, 1991; Rapaport et al., 2015).

Several physiological measurements were carried out over the course of this experiment, for early detection of the parasite. None of the measurements revealed any differences between treatments in the early stage of the parasitism. This finding is similar to those of previous studies (Barker et al., 1996; Hibberd et al., 1998), which demonstrated that the maintenance of plant functioning during parasite development is essential, due to the parasite’s need for the energy and sugar produced by the host. Therefore, a decrease in plant functioning occurs only after the parasite completes its life cycle. Although Ortiz-Bustos et al. (2016) reported inconsistent changes in chlorophyll content, in this study, no changes were seen in chlorophyll content. Moreover, no differences in the chlorophyll-related waveband (650–700 nm) were noted between the infected and non-infected plants.

As an obligate parasite, *O. cumana* gets all of its essential nutrients from its host. Furthermore, parasite development on host plant roots reduces the mineral content of the host plant (Westwood, 2013). In this experiment, reductions in the levels of several minerals (including essential macro- and microelements) were observed. The most significant reduction in the infected plants was found in levels of potassium, a very mobile element related to plant osmotic regulation. All of the other elements examined are known to be mobile or semi-mobile in the plant and can move rapidly to a strong sink. Carbon and hydrogen, however, showed an opposite pattern between treatments during the experiment; their levels increased (**Figure 3**). The relative increase in carbohydrate content can be explained by the increased demand for carbohydrates in the newly created sink, which leads to higher production of carbohydrates in the leaves (Nandula et al., 2000). Similar results were demonstrated in *Phelipanche aegyptiaca* parasitism of carrot and tomato (Nandula et al., 2000; Shilo et al., 2016). Another possible explanation is that a decrease in mineral content in the tissue leads to relative increases in the carbohydrate content. The results of this study contradict results of a previous study concerning the effect of *O. cumana* parasitism on mineral concentrations in sunflower leaves. However, that study dealt with *O. cumana* only after inflorescence emergence (Alcántara et al., 2006), while the current work focuses on earlier stages of the parasitism.

The most noteworthy change found to be caused by *O. cumana* infection was the change in the relative amount of air space in the leaf mesophyll. This change in the mesophyll tissue was detected almost exactly at the predicted time for the first *O. cumana* attachment, according to Eizenberg et al. (2012b). Previously, changes in mesophyll tissue have been recorded in response to salinity stress and water deficit (Delfine et al., 1998; Rapaport et al., 2015). These studies demonstrated reduction in the mesophyll content and organization, caused by stress. Even earlier research demonstrated mesophyll disintegration caused by osmotic stress (Giles et al., 1974), stress known to induce abscisic acid metabolism in the plant (Zhu, 2002). Moreover, plant parasites are known to increase abscisic acid content in the host plant (Frost et al., 1997). This study demonstrates an instant relationship between *O. cumana* attachment and mesophyll disintegration. The observed damage to the mesophyll tissue can be explained by changes in mineral availability, primarily potassium, due to the new sink demands. Potassium deficit, due to it mobility, can lead to dramatic changes in the mesophyll structure (Zhao et al., 2001; Battie-Laclau et al., 2014).

Correlation between leaf mineral content and reflectance at specific wavelengths indicate that the changes in reflectance in the SWIR range are highly correlated with changes in levels of potassium, phosphorus, magnesium, and boron in the leaf. These related changes are known from previous work on mineral deficits in leaves (Al-Abbas et al., 1972). In addition, the statistical analysis revealed a high correlation between nutrient content and reflectance in the 650–750 nm range. However, no changes in reflectance related to *O. cumana* parasitism were observed within this range. The statistical analysis apparently revealed differences that were underway, but not yet manifested in a change in reflectance. Also, reflectance within this range is known to correlate closely with chlorophyll content and fluorescence (Ercoli et al., 1993) but no differences in chlorophyll content were noted in this study.

Changes in leaf structure and mesophyll structure, in particular, are known to be related to changes in far-NIR (800–1300 nm) reflectance (Johnson et al., 2005; Ollinger, 2011). Although PLS-R analysis revealed no differences in this area, the statistical analysis of the reflectance at these wavelengths revealed developing difference in this area after *O. cumana* attachment. The difference in reflectance between the *O. cumana*-infected and non-infected control plants increased after attachment, from 1% after attachment to 2.5% after the emergence of the *O. cumana* inflorescences. The different treatments had a differential effect on the correlation between reflectance in the NIR range and leaf air space only after *O. cumana* attachment, indicating that reflectance within this range is indicative of *O. cumana* parasitism. Hence, use of these specific wavelengths can lead to good early detection of *O. cumana* in sunflower. Moreover, data from the control samples of resistant cultivar demonstrated that the presence of parasite seeds in the soil had no effect on leaf spectral, physiological and mineral content, nor on leaf mesophyll structure. Furthermore, the use of resistant variety support our assumption that the differences between the infected and non-infected sunflower are due to the parasitism and not from other physiological stresses.

## Conclusion

Effective management of *O. cumana* requires the application of herbicide at the early stage of parasitism, at which point no external effects are visible. This study stresses two points. First, *O. cumana* causes rapid changes in leaf nutrient content, which lead to immediate changes in the structure of the leaf mesophyll of the host. Second, these changes can be observed using hyperspectral equipment. Combining the thermal model developed by Eizenberg et al. (2012b) with hyperspectral imaging should enable detection of the spatial distribution of the parasite at an early stage of parasitism. Future work should focus on how this integrated model can be applied in the field.

## Author Contributions

AC: performed greenhouse measurements, data analysis, and writing the manuscript. TR: performed greenhouse spectral measurements and PLS-R analysis. TG: performed anatomic cross-sections. AK: support in data analysis and writing. HE: head of the lab on parasitic weeds. SR: support in data analysis and writing. JE: support in data analysis and writing.

## Conflict of Interest Statement

The authors declare that the research was conducted in the absence of any commercial or financial relationships that could be construed as a potential conflict of interest. The reviewer AVA and handling Editor declared their shared affiliation, and the handling Editor states that the process met the standards of a fair and objective review.
